# Effect of heat treatment with different heat transfer modes on the polymerization of tosylate-doped poly(3,4-ethylenedioxythiophene) films

**DOI:** 10.1038/s41598-022-13510-9

**Published:** 2022-06-07

**Authors:** Hyeong Jun Kim, Jei Gyeong Jeon, Ju Hwan Lee, Ju Hyeon Kim, Junho Lee, Gilyong Shin, Tae June Kang

**Affiliations:** Department of Mechanical Engineering, Inha University, 100 Inha-ro, Michuhol-gu, Incheon, 22212 Republic of Korea

**Keywords:** Engineering, Materials science

## Abstract

In this work, tosylate-doped poly(3,4-ethylenedioxythiophene) (PEDOT:Tos) films are prepared by thermally assisted oxidative polymerization either on a hot plate or in a convection oven. The main difference between these heat treatments is the way heat is transferred (conduction or convection) during polymerization. The surface morphology and structure, doped state, chemical composition, and the changes in the physical and chemical properties of the differently heat-treated films are analyzed using various instrumental methods. The hot plate-treated films exhibit a smooth and dense surface morphology with a low root-mean-square roughness of ~ 5 nm. The films have a quinoid-prevalent thiophene structure with a high electrical conductivity of 575 S/cm. By contrast, the oven-treated films show a rough and porous morphology with a surface roughness ranging from 30 to 80 nm depending on the scanning area, which yields high absorption capacity of more than 90% in the near-infrared range. The oven-treated films show a benzenoid-prevalent structure that provides relatively low electrical conductivity of 244 ± 45 S/cm. As a demonstration of these noticeable changes, PEDOT:Tos films are examined as a photothermal conversion layer to convert light energy to thermal energy, which is converted to electrical energy using a thermoelectric device by covering the films on the device.

## Introduction

With their inherently flexible nature and tunable electrical properties, conducting polymers are an integral part of emerging flexible and functional electronics, such as rollables, photovoltaic cells, and wearables^1–6^. Among the numerous conductive polymers implemented in such devices, the polythiophene derivative of poly(3,4-ethylenedioxythiophene), or PEDOT, has shown significant promise owing to its desirable flexibility, transparency, and remarkable stability under ambient conditions^7–11^. Solution processability suitable for low-cost, large-scale production provides economic advantages for commercialization.

As a conjugated polymer with C–C and C=C bonds, PEDOT carries positive charges along the polymer chain and exhibits aromaticity with the modest electrical conductivity of a semiconductor. The conductivity of PEDOT can be increased by doping with an oxidizing agent or a p-type dopant, which renders the doped PEDOT more hole conductive. For a counter anion in PEDOT doping, tosylate (Tos) has been used to produce PEDOT:Tos films with high electrical conductivity^12–14^. In contrast to the hydrophilic dopant of polystyrene sulfonate (PSS) for PEDOT:PSS^15,16^, the hydrophobic nature of PEDOT:Tos improves the stability against humidity, which widens the applicability of the conductive polymer to electronics, such as functional electrodes, displays and organic thermoelectric devices^17–19^.

To implement PEDOT:Tos films in such applications, efforts for improving the properties of PEDOT:Tos have focused on optimizing the composition of the precursor mixtures and synthesis methods^20,21^. Oxidative polymerization is involved in the synthesis of PEDOT, which can be assisted by heat treatment to accelerate the polymerization rate of 3,4-ethylenedioxythiophene (EDOT). The tosylate anion, as a charge balancing counter ion, then stabilizes the positively charged PEDOT chain. The synthesis procedure for the oxidative polymerization of PEDOT:Tos involves coating a substrate with a mixture of the EDOT monomers and an oxidative solution of iron(III) tris-toluenesulfonate (Fe(Tos)_3_). Polymerization proceeds by subjecting the precursor coating to an elevated temperature for a time. During heat treatment, the Fe^3+^ oxidant in the iron-tosylate oxidizes EDOT, forming cationic radicals, which consecutively form a dimer. Similarly, the dimers are oxidized and polymerized in PEDOT. Finally, unreacted precursor molecules and any by-products are removed from the precursor coating by rinsing with a solvent, such as ethanol. A more detailed description of the polymerization process is provided in Supporting Information 1.

In the synthesis procedure of PEDOT:Tos, heat treatment can be involved to initiate and accelerate the polymerization reactions^22^. On the other hand, the use of different heat treatment apparatuses may result in PEDOT:Tos films with different morphologies and properties because of their difference in the heat transfer mode to proceed with polymerization. In this study, PEDOT:Tos films were prepared by the thermally assisted oxidative polymerization of EDOT monomers either on a hot plate or in a convection oven. The changes in physical and chemical properties for the differently heat-treated films were thoroughly examined using various instrumental methods.

## Results and discussion

### Morphology and optical property of PEDOT:Tos films

PEDOT:Tos films were prepared by the thermally assisted oxidative polymerization of EDOT monomers in the presence of Fe(Tos)_3_. The effect of different heat transfer modes on the morphology and properties of the resulting films were compared by fixing the other conditions for the film synthesis, such as the precursor composition, coating method, temperature, and time for the heat treatments.

**Figure 1:**
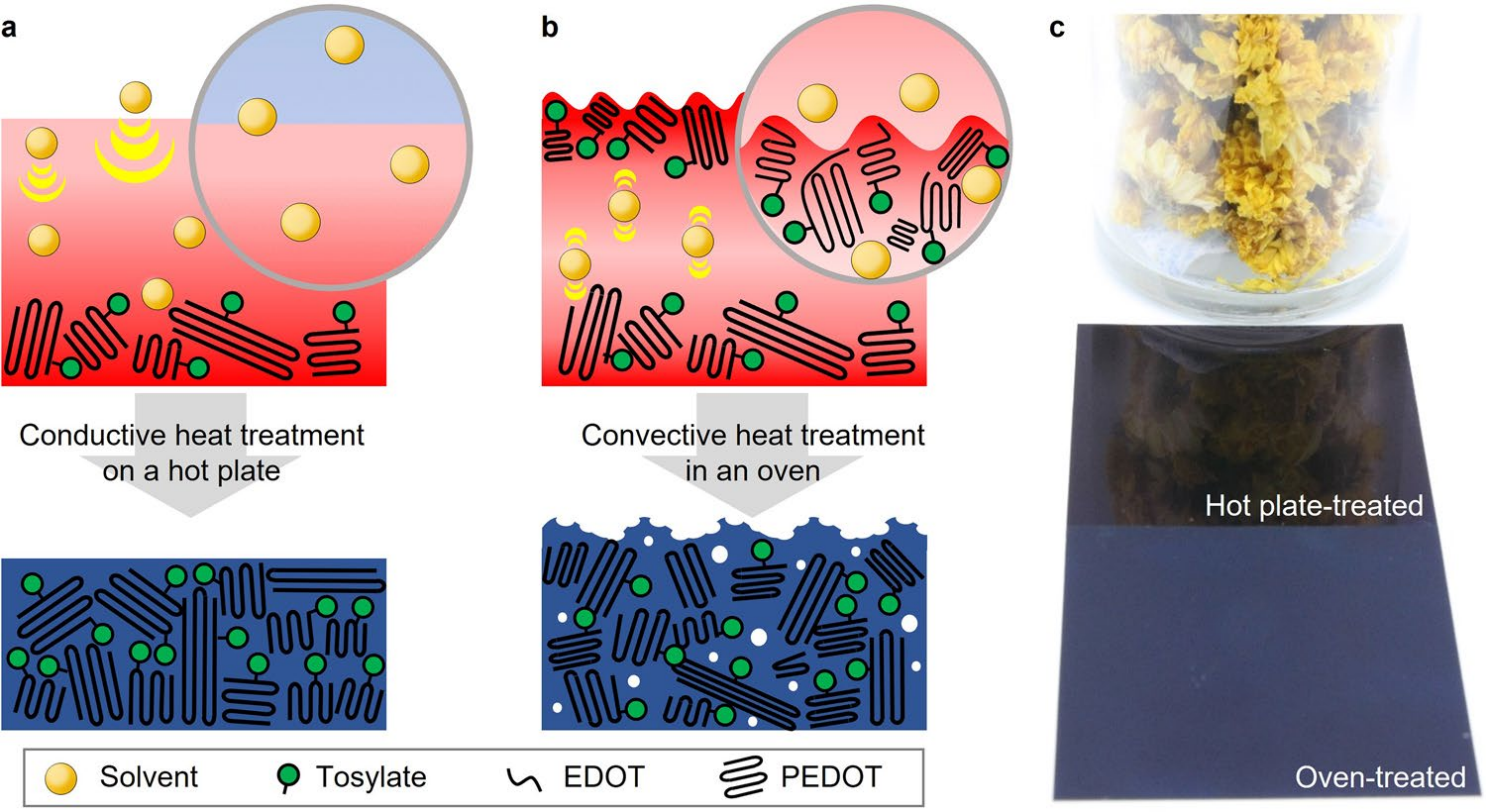
(**a**) and (**b**) are the schematics of the difference in the morphology of the PEDOT:Tos films depending on the heat transfer modes in the heat treatment of the precursor coating. (**c**) Optical image of the PEDOT:Tos films. The hot plate-treated film reflects an object placed around it on a smooth surface, whereas the oven-treated film does not because of its roughened surface.

Figures 1a and b are the schematics to highlight the differences in the morphology of the PEDOT:Tos films according to the heat treatment of the precursor coating using either a hot plate or a convection oven, respectively. The details for the materials and the preparation of the precursor coating are provided in the Materials and methods section. The most significant difference between using a hot plate or a convection oven lies in the mode of heat transfer for polymerization. Heat treatment using a hot plate is driven by heat conduction in the thickness direction of the precursor coating from the bottom in contact with a heat source to the top surface subjected to open air. Polymerization initiates at the bottom and successively proceeds to the top portion with a directional flux of solvent evaporation, as depicted schematically in Fig. 1a. When the unreacted precursor was washed away with ethanol, and the substrate was dried, the thermal process may produce a dense structure and a smooth surface of the films.

For the oven treatment, however, the top portion of the precursor coating is the first to be polymerized by evaporating the solvent in that region and densifying the polymer skin layer. Because the densified layer is typically resistive to heat transfer and barely allows the facile evaporation of solvents, they might act as a diffusion barrier to prevent solvent evaporation from the precursor coating to the surrounding. This can result in a dimpled texture on the surface of films with blisters and bubbles by outshooting the solvent in the precursor coating through the densified layer during heat treatment. Therefore, the oven treatment produces a porous structure and high surface roughness of the films, as illustrated in Fig. 1b. This phenomenon is termed the ‘skin effect’ in this work.

Figure 1c shows an optical image of PEDOT:Tos films prepared on a PET substrate using a hot plate and a convection oven. The hot plate-treated film reflects an object (a vase with flowers) placed around it on the smooth surface, whereas the oven-treated film did not owing to its roughened surface.

**Figure 2:**
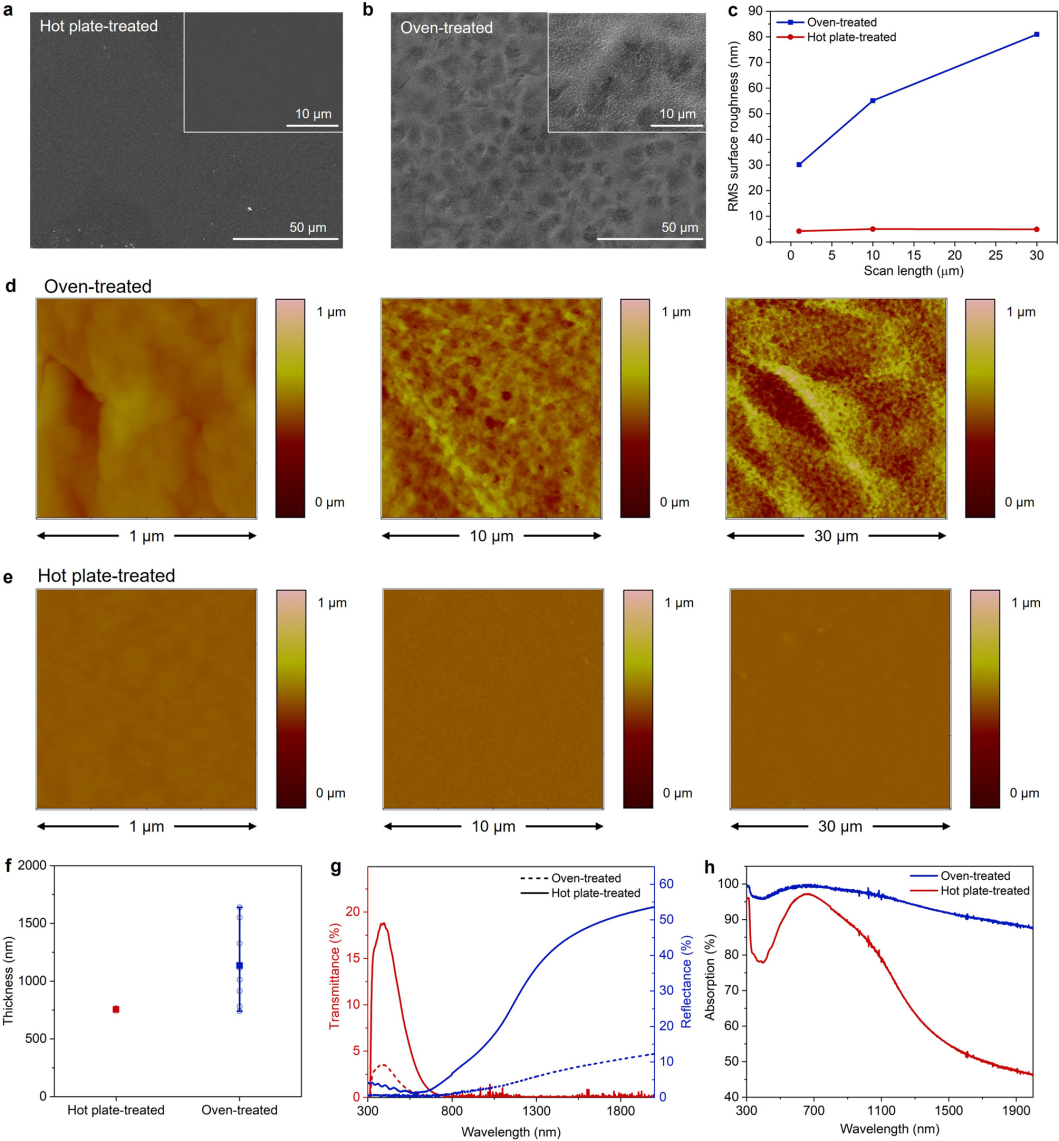
SEM images of (**a**) the hot plate-treated and (**b**) oven-treated PEDOT:Tos films. (**c**) The RMS surface roughness of the films with respect to the side length of the scan area. AFM images of (**d**) oven-treated and (**e**) hot plate-treated PEDOT:Tos films for the varying scan areas. (**f**) Thickness of the PEDOT:Tos films. (**g**) Transmittance and reflectance spectra and (**h**) the absorption spectra for the films in wavelength ranges, including visible and NIR regions.

The differences in morphology of the surfaces of the PEDOT:Tos films were examined by scanning electron microscopy (SEM). While the hot plate-treated film showed a smooth and flat surface (Fig. 2a and the inset in the figure for the enlarged image), the oven-treated film exhibited a highly rough surface with a crater-like morphology (Fig. 2b). As shown in the enlarged image of the inset in Fig. 2b, close observation showed that the surface comprised a rough morphology of blisters and bubbles on the dimpled texture. The root mean square (RMS) surface roughness over square areas with sides of 1, 10, and 30 μm was analyzed quantitatively by atomic force microscopy (AFM), as shown in Fig. 2c. AFM images for each measurement are also provided in Fig. 2d and e. As shown in Fig. 2c, the root mean square (RMS) surface roughness was as low as ~ 5 nm for the hot plate-treated film. The roughness remained relatively constant for the varying scan areas, indicating smooth and flat morphology was achieved on a whole surface area of the hot plate-treated film. The oven-treated film, however, showed a much higher roughness compared to the hot plate-treated film. In particular, the roughness changed from 30 to 80 nm as the side length of the scan area increased from 1 to 30 μm due to the roughness.

The thickness of the PEDOT:Tos films was also determined by AFM by measuring the depth of a groove scratched in the films (Fig. 2f) (See also AFM images of the measurements in Supporting Information 2). The hot plate-treated PEDOT:Tos films showed an average thickness of ~ 750 nm with a small standard deviation of 13 nm. By contrast, the oven-treated film showed a thickness of ~ 1150 nm with a large standard deviation of ~ 261 nm, as expected from its highly rough and porous surface morphology. The increase in the roughness and thickness can be explained by the skin effect pronounced in the oven-treated films, as shown schematically in Fig. 1b.

The difference in the morphology for the differently heat-treated PEDOT:Tos films can affect the optical property of the films significantly. The transmittance and reflectance spectra of the specimens were acquired in the wavelength range from 300 to 2000 nm using ultraviolet–visible (UV–Vis) spectroscopy (Fig. 2g). The absorption spectra were then calculated using the measured transmittance and reflectance data (Fig. 2h). As shown in Fig. 2g, both PEDOT:Tos films exhibited a transmittance to some extent in the visible range, but the oven-treated film showed a much lower transmittance of 3.5% at 450 nm, almost 80% lower than that of the heat-treated (18.8%). Both films showed an almost zero transmittance in the NIR range over 800 nm. For the reflectance, the oven-treated films showed a low reflectance of 10% or less in the NIR range, whereas the hot plate-treated showed increasing and relatively high reflectance in the range. These transmittance and reflectance results suggest that the oven-treated PEDOT:Tos film had higher light absorption capacity in both the visible and NIR regions than the hot plate-treated film, as shown clearly in Fig. 2h. To examine the effect of film thickness on the difference in absorption capacity, the absorption coefficient that is the absorbance normalized to the sample thickness was calculated. As can be seen in Figure S3 in Supporting Information, the hot plate-treated PEDOT:Tos film exhibited a slightly higher absorption coefficient in the measured range than the oven-treated film. The higher light absorption capacity of the oven-treated film thus can be attributed to its relatively thick thickness, rough surface and porous morphology.

### Chemical structure and doped state of PEDOT:Tos films

**Figure 3:**
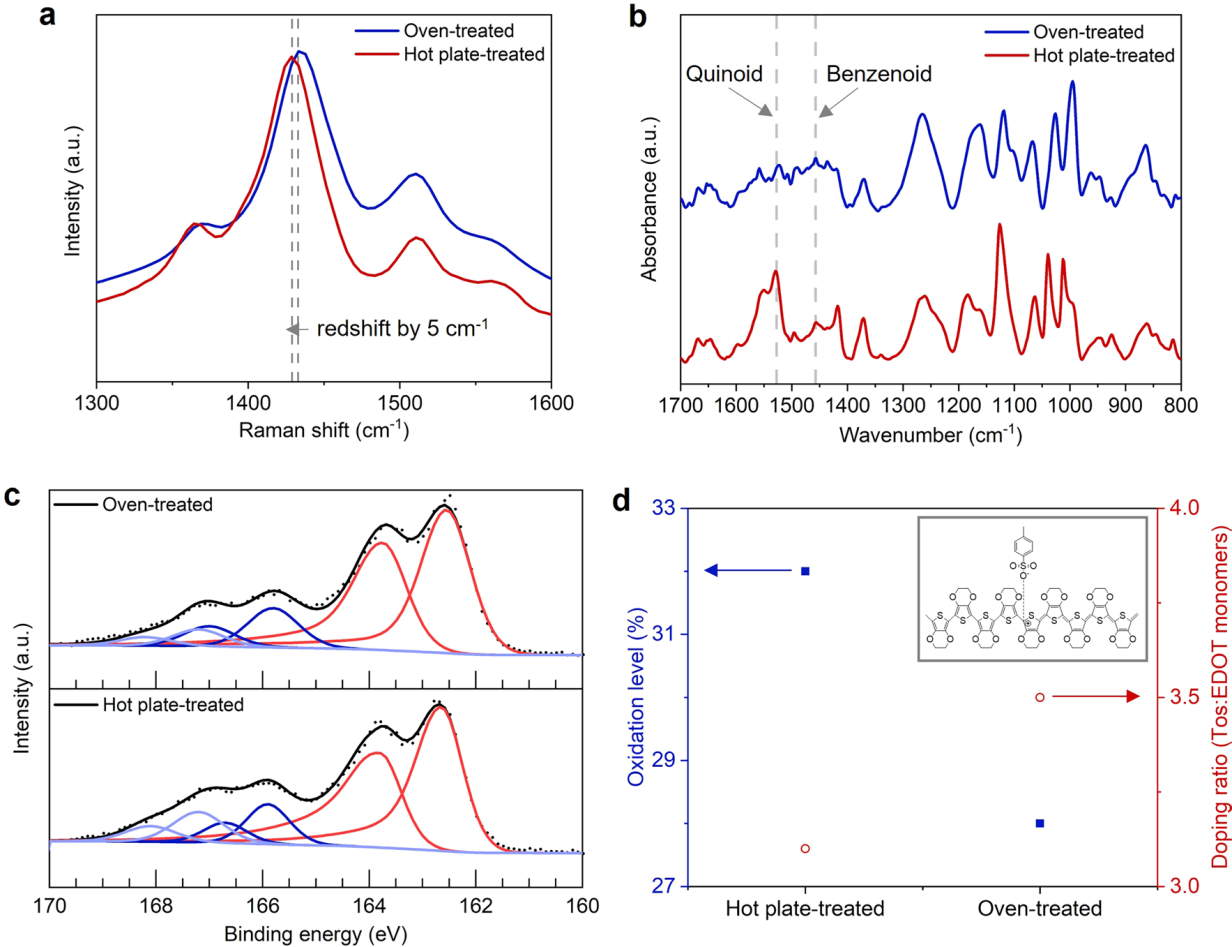
(**a**) Raman analysis of the chemical structure of the films by comparing the vibrational modes of molecules in the PEDOT chain. (**b**) ATR FT-IR analysis of the films and (**c**) XPS analysis for the chemical composition of the PEDOT:Tos films and (**d**) the doped state of tosylate ions into PEDOT.

To investigate the skin effect on the physical and chemical properties, PEDOT:Tos films were analyzed by various instrumental methods. Raman spectroscopy was first carried out to examine the chemical structure of the films by comparing the vibrational modes of molecules in the PEDOT chain (Fig. 3a). Three major bands were detected in both specimens around 1365 cm^-1^, 1432 cm^-1^, and 1511 cm^-1^, which were assigned to the symmetric C_β_–C_β_ and C_α_=C_β_ stretching and the asymmetric C_α_=C_β_ stretching in thiophene ring, respectively, as reported previously^23,24^ for the structural fingerprint of PEDOT. On the other hand, the Raman band of the symmetric C_α_=C_β_ stretching for the hot plate-treated film (1428 cm^-1^) was 5 cm^-1^ lower than that of the oven-treated PEDOT:Tos film (1433 cm^-1^). The quinoid structure with a single C_α_–C_β_ bond exhibits a lower force constant because of the delocalizing π-electron involved in the bond than the benzenoid structure of the C_α_=C_β_ bond in the thiophene ring of PEDOT^25^. In this regard, the redshift might have been caused by the higher composition of quinoid structures in the hot plate-treated PEDOT:Tos film compared to the oven-treated film.

Attenuated total reflection Fourier transform-infrared (ATR FT-IR) spectroscopy was carried out to investigate the relative composition of benzenoid and quinoid moieties in thiophene, as shown in Fig. 3b. While the characteristic peak positions of PEDOT:Tos detected were consistent with the literature^21,26,27^, the quinoid-benzenoid composition was compared from the intensity ratio of the asymmetric C_α_ = C_β_ stretching of the quinoid (centered at 1529 cm^-1^) and benzenoid (peak centered at 1457 cm^-1^) structures. As can be seen in the figure, the quinoid structure was pronounced in hot plate-treated PEDOT:Tos film with the intensity ratio of 2.37:1, whereas the benzenoid structure was dominant for the oven-treated film with 1:1.16. These results are in good agreement with the Raman spectra shown in Fig. 3a.

The chemical composition and the doped state of tosylate ions into PEDOT were analyzed by X-ray photoelectron spectroscopy (XPS) of the two different sulfur atoms present in PEDOT:Tos (Fig. 3c). The XPS signal attributed to the S atom in tosylate was observed from 165 to 169 eV, whereas the S in the thiophene ring of PEDOT was observed at lower binding energies from 161 to 165 eV. In particular, the XPS peaks at 162.65 and 163.85 eV in Fig. 3c showed the spin–orbit peaks of the S atom (S_2p_) in the thiophene ring of PEDOT. The peaks at 165.9 and 167.2 eV were assigned to S_2p_ in the tosylate ions that are electrostatically bonded to the PEDOT chains. These characteristic peaks of the PEDOT:Tos films were in accordance with the previous reports^28,29^, indicating the films had been synthesized successfully by the present heat treatment condition. The doping ratio of tosylate ions into PEDOT chains was estimated by comparing the ratio of the area covered by the thiophene S_2p_ peaks to that of tosylate in the binding energy of S_2p_^30,31^. As shown in Fig. 3d, the hot plate-treated films showed a relatively higher oxidation level of 32%, compared to the oven-treated films (28%). Correspondingly, the doping ratio of tosylate ion to EDOT monomers for the hot plate was calculated to 1:3.1, which is close to the ideal doping state of one positive charge per three monomer units^32–34^, while the oven-treated PEDOT:Tos showed lower doping ratio 1:3.5.

The major electrical conduction pathways of the PEDOT:Tos film are the charge transport along the PEDOT chains and along the π- π stacking between the chains^35^, in which the doping level determines the density of charge carriers. In addition, quinoid predominant structure in thiophene can further promote π-orbital overlap compared to aromatic structure. Thus, the higher doped state and the relatively higher composition of quinoid structures in the hot plate-treated films could lead to higher electrical conductivity compared to that of the oven-treated films. Actually, the electrical conductivity of hot plate-treated films was measured to be 575 S/cm using a four-point probe with the thickness data measured in Fig. 2d, which is approximately two times higher than the oven-treated films (244 ± 45 S/cm).

### Photothermal conversion applications of the PEDOT:Tos films

**Figure 4:**
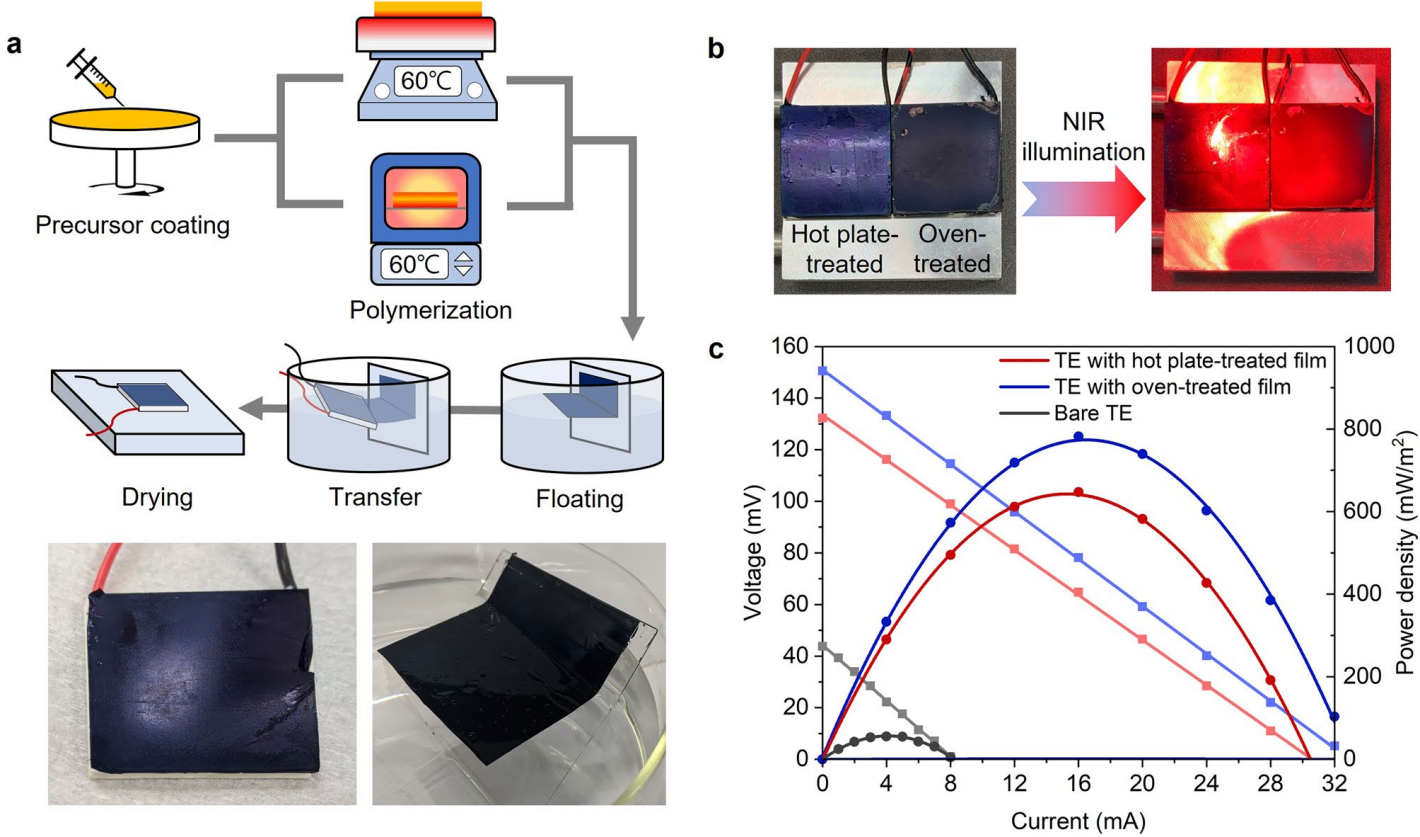
(**a**) Experimental procedure to fabricate the TE device with the PEDOT:Tos overlayers for photothermal conversion. Optical images of the freestanding PEDOT:Tos film on water and the prepared TE device covered with the film are shown below the figure. (**b**) Evaluation of the performance of photothermal conversion performance of the films by measuring an output power from the TE devices with the PEDOT:Tos overlayers.

As shown in Figs. 2, 3, the difference in the heat transfer modes had a significant influence on the morphology and the related optical and electrical properties of the PEDOT:Tos films produced. To determine if these changes could be useful for applications, we investigated the photothermal conversion performance of the PEDOT:Tos films to convert light energy to thermal energy, which was, in turn, converted to electrical energy via a thermoelectric (TE) device by coating with the films. Figure 4a shows the experimental procedure to fabricate the TE device with the PEDOT:Tos overlayer for photothermal conversion. The PEDOT:Tos film prepared with the different heat treatments was deposited onto a hydrophilic substrate, such as quartz, and the specimen was then immersed in water. The water molecules penetrated between the film and hydrophilic substrate, which induced spontaneous delamination by allowing the film to float on the water surface. The floating PEDOT:Tos film was transferred to the TE device by scooping up the film out of the water slowly. Finally, the TE device with the PEDOT:Tos overlayer was dried under ambient conditions. Optical images of the freestanding PEDOT:Tos film on water and the prepared TE device covered with the film are shown in Fig. 4a.

The effectiveness in using the PEDOT:Tos films as a photothermal conversion layer was evaluated by measuring the output power from the TE devices with the PEDOT:Tos overlayer under NIR light illumination with a power density of 0.1 W/cm^2^ at 1200 nm. To measure the power generated, the top surface of the TE device was illuminated with NIR light, and the temperature on the bottom was maintained at 23 °C by placing it on a fluid-cooled plate connected to a cold thermostatic bath (see Fig. 4b). As shown in Fig. 4c, the TE device covered with the hot plate-treated PEDOT:Tos film provided a power density of 642.7 mW/m^2^, which was approximately 11 times higher than that of the bare TE. This improvement in the power generation was attributed to the increased ∆T with the PEDOT:Tos overlayer by increasing an open-circuit voltage (V_oc_) from ~ 44 mV for the bare TE to 132 mV for the TE with the overlayer. The output power was further improved to 773.7 mW/m^2^ with a 13% increase in V_oc_ to 151 mV by implementing the oven-treated PEDOT:Tos film. The rough and porous morphology of the oven-treated film led to this improvement in power generation because of the higher absorption ability of NIR light, as well as the higher efficiency in photothermal conversion (Supporting Information 4).

## Conclusion

In this work, we examined the effects of the heat transfer mode of the heat treatment on the morphology and the properties of conducting PEDOT:Tos films. The surface morphology and structure, doped state, chemical composition, and the changes in the physical and chemical properties of the differently heat-treated films were analyzed using various instrumental methods. The hot plate-treated film showed a smooth and dense surface morphology with quinoid-prevalent thiophene rings along the polymer chain, and high electrical conductivity. On the other hand, the oven-treated film showed a rough and porous morphology with a benzenoid-prevalent structure, and a high light absorption capability, particularly in the NIR range. In conclusion, these results suggest that close attention should be paid to the choice of heat treatment apparatus because the morphology and properties of the films are affected significantly by the heat transfer mode for thermally assisted polymerization.

## Materials and methods

### Preparation of the PEDOT:Tos films

The precursor solution was first prepared by mixing a 40 wt.% solution of iron(III) p-toluene sulfonate (CLEVIOS™ C-E 40, Heraeus) in ethanol, 3,4-ethylenedioxythiophene (EDOT 97%, Sigma-Aldrich), and pyridine (Pyridine anhydrous 99.8%, Sigma-Aldrich) at a volume ratio of 1.0 ml : 33.2 μL : 13.8 μL. A 196.5 μL sample of poly(ethylene glycol)-block-poly(propylene glycol)-block-poly(ethylene glycol) (PEG-PPG-PEG, average Mn ~ 5,800, Sigma-Aldrich) was also added to the solution as a growth template for PEDOT:Tos chains. The component and composition of the precursor solution used here were those reported in the literature^36^_._ The precursor solution was then spin-coated onto a substrate, such as polyethylene terephthalate (PET), at 400 rpm for 60 s. The specimen was heat-treated using a hot plate or convection oven at 60 °C for one hour to polymerize the precursor coating. Finally, the PEDOT:Tos films were produced by washing away the unreacted precursor and by-products with ethanol, followed by drying under ambient conditions.

### Fabrication of PEDOT:Tos combined thermoelectric device

To transfer the photothermal layer onto the thermoelectric device, the PEDOT:Tos precursor solution was first coated on a quartz substrate with a size of 7 × 7 cm^2^. For the hot-plate treated PEDOT:Tos, the heat treatment was applied on a hot plate at 60 °C for one hour, followed by ethanol washing and immersing in a water bath. For the oven-treated PEDOT:Tos, the precursor solution was coated onto another quartz substrate. The heat treatment was performed in a convective oven at 60 °C for one hour to form a highly porous PEDOT:Tos photothermal layer. After the polymerization, the PEDOT:Tos film attached on the substrate was immersed in a water bath and the film floated on the surface of the water. Then the film was transferred to the surface of thermoelectric element, drying under an ambient atmosphere. To measure the power generated, the top surface of the TE device was illuminated with NIR light at 1200 wavelength, and the temperature of the bottom side was maintained at 23 °C by placing it on a fluid-cooled plate connected to a cold thermostatic bath. I–V curve was obtained with a potentiostat (Corrtest, Wuhan CorrTest Instruments Corp.).

### Instrumental analysis

The surface morphology, roughness, and thickness of the PEDOT:Tos specimen were evaluated by AFM (Nanoscope Multimode IVa, Bruker). Optical properties, such as transmittance and reflectance, were examined by UV–vis spectroscopy (Varian Cary 500 Scan UV–Vis spectrophotometer, Varian). Raman spectroscopy (Ramanforce, Nanophoton) was used to analyze the chemical structure of the films with a laser wavelength of 532 nm. XPS (K-Alpha, Thermo scientific) was performed using a monochromated Al Kα X-ray source to examine the chemical composition of the films and the doped state of tosylate ions in PEDOT. ATR FT-IR spectroscopy (VERTEX 80 V, Bruker) was used to quantify the ratio of benzenoid and quinoid moieties in thiophene of the PEDOT:Tos films.

## Supplementary Information

Supplementary Information.

## Data Availability

All data are available from the corresponding author on reasonable request.
