# Supplementary material for: Effect of heat treatment with different heat transfer modes on the polymerization of tosylate-doped poly(3,4-ethylenedioxythiophene) films
Source: Sci Rep. 2022 Jun 7;12:9386. doi: 10.1038/s41598-022-13510-9 (PMC9174293; doi:10.1038/s41598-022-13510-9)
Supplement: Supplementary file 1 — Supplementary Information. [file 41598_2022_13510_MOESM1_ESM.pdf]

Supporting Information

Effect of heat treatment with different heat transfer modes on the polymerization of tosylate-doped poly(3,4-ethylenedioxythiophene) films

Hyeong Jun Kim, Jei Gyeong Jeon, Ju Hwan Lee, Ju Hyeon Kim, Junho Lee, Gilyong Shin, and Tae June Kang*

Department of Mechanical Engineering, Inha University, 100 Inha-ro, Michuhol-gu, Incheon, 22212, Republic of Korea

* tjkang@inha.ac.kr

1. Oxidative polymerization of PEDOT:Tos

PEDOT:Tos can be produced by mixing the 3,4-ethylenedioxythiophene (EDOT) monomers and an oxidative solution of iron(III) tosylate. The EDOT monomer was oxidized by Fe^{3+}, and EDOT radical was formed from a proton and the resulting Fe^{2+}. A long chain of PEDOT was polymerized as the oxidative reaction continues, as shown schematically in Figure S1. Iron(III) tosylate is generally preferred because of its low effective oxidation strength, which causes a slow reaction rate for the polymerization of PEDOT. This feature enables the production of long PEDOT chains with extended and uninterrupted conjugation, thereby improving the hole transportation characteristics. To improve the conductivity further by reducing the rate of this oxidative polymerization, various compositions for the precursor mixture have been extensively explored with the addition of inhibitors, such as pyridine (used in this work), imidazole, or N-methyl-2-pyrrolidone to the mixture. Such basic inhibitors in the

precursor attract the free protons released from the oxidation process in the reaction media, which can reduce the oxidative polymerization rate of EDOT and prevent acid-initiated polymerization that results in poor conjugation.

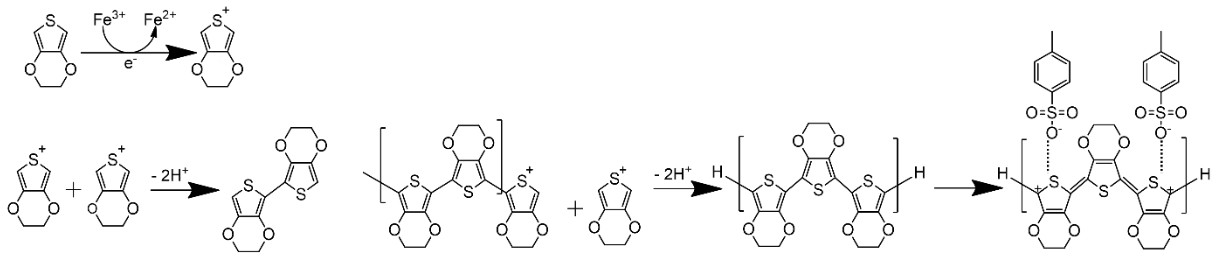

Figure S1. Schematic representation of oxidative polymerization of PEDOT:Tos

2. Thickness measurements of PEDOT:Tos films using AFM

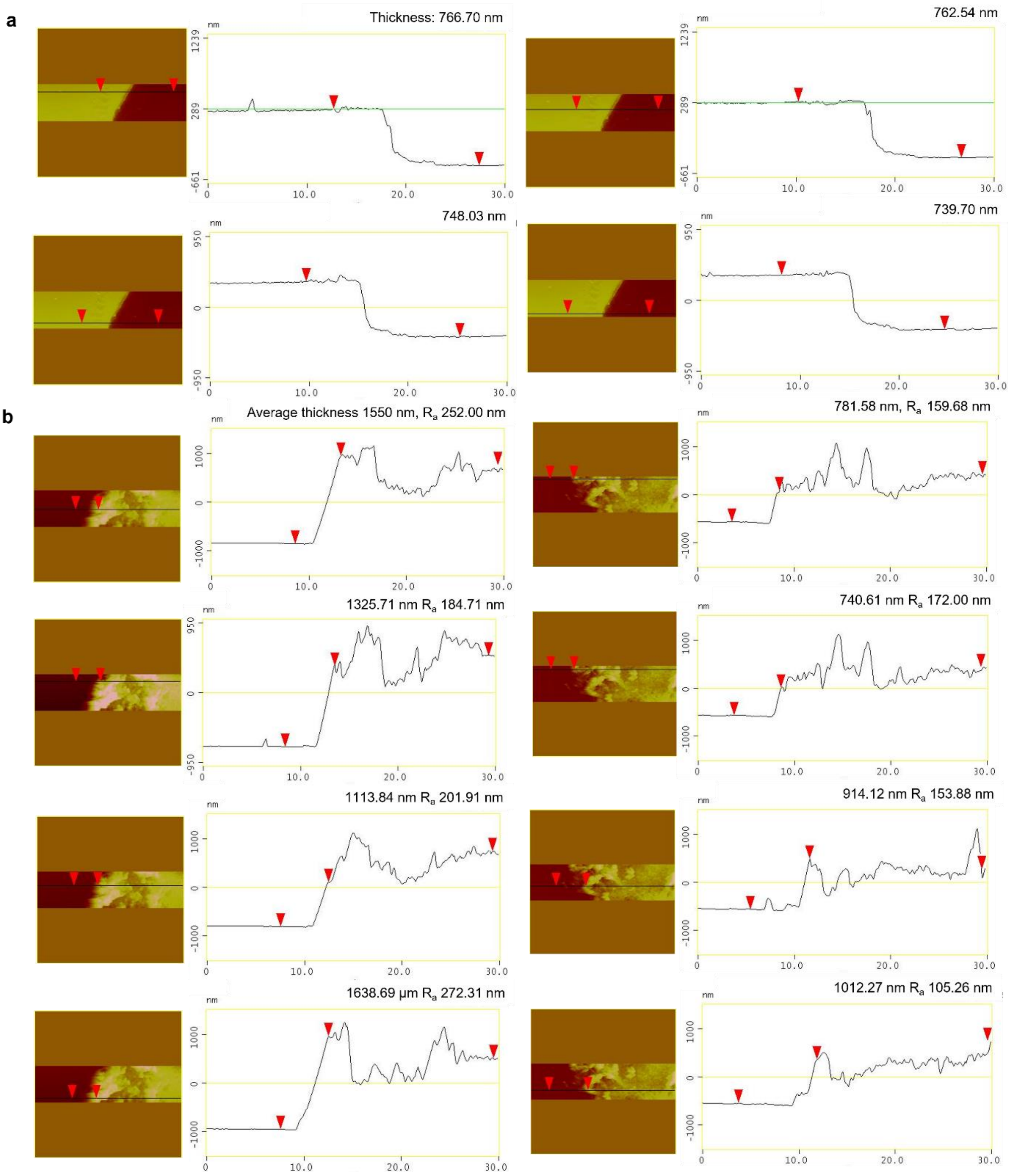

Figure S2. Thickness measurements of (a) hot plate-treated and (b) oven treated-PEDOT:Tos films. The thickness of oven-treated-PEDOT:Tos films was estimated by adding the average roughness to the thickness of the thinnest part of the film.

3. Calculated absorption coefficient of PEDOT:Tos films

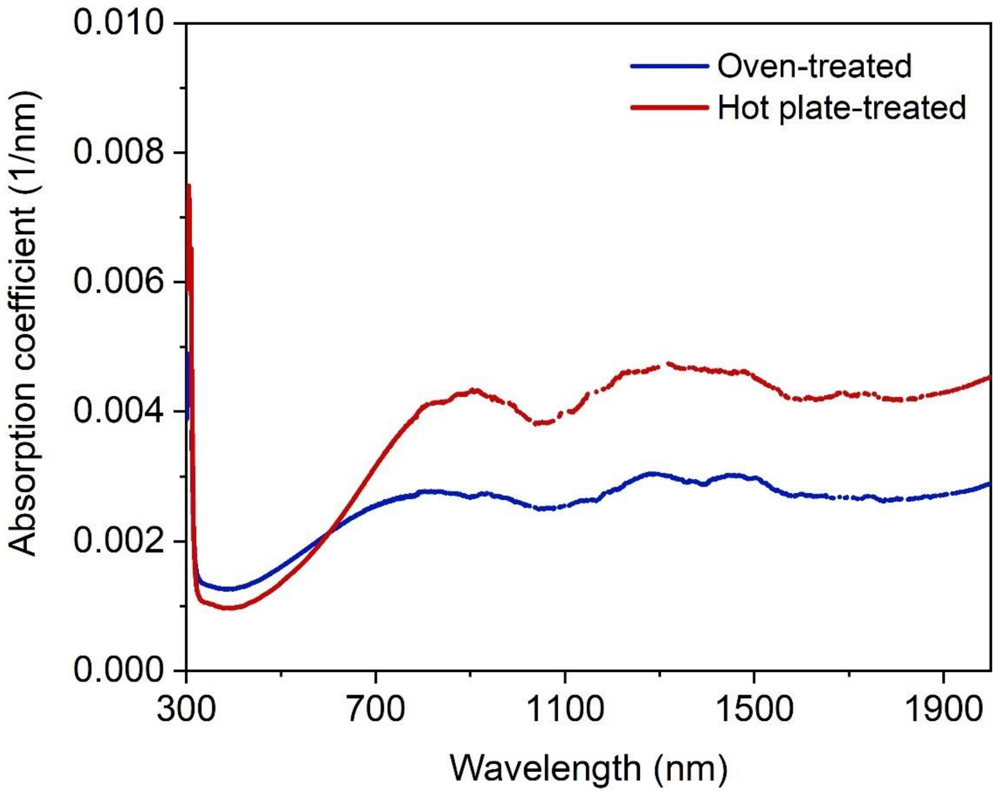

Figure S3. Absorption coefficient spectra for the PEDOT:Tos films

4. Photothermal conversion efficiency of PEDOT:Tos films

PEDOT:Tos films were prepared on a PET substrate to evaluate their photothermal conversion efficiency. To prepare the measurement setup, the film was cut into 2×2 cm^2 size and floated in air by supporting the edges of it with a thermally insulating rectangular frame. The specimen was irradiated with IR light with a power density of 950 W/m^2. The increase in the temperature of the film was monitored using a K-type thermocouple attached to the top surface of the film. The photothermal conversion efficiency was calculated based on the energy balance for thermal equilibrium, as follows:

$$\sum mc\frac{dT}{dt} = Q_{Photothermal} - Q_{loss} - Q_{sub} \qquad (1)$$

$$Q_{Photothermal} = I_0\left(1 - 10^{-A(\lambda)}\right) * \eta_{PCE}, \; Q_{loss} = hA(T - T_\infty) \qquad (2)$$

where η_{PCE} is the photothermal conversion efficiency; h is a heat transfer coefficient; A is the surface area of the film; Q_{sub} is the amount of heat absorbance at the PET substrate; I_0 is an input light power; $A(\lambda)$ is the absorbance at specific wavelength λ; Q_{loss} is the amount of heat transferred to the atmosphere, and mc_p is the thermal capacitance of the film.

At steady state, dT/dt should be zero, then, the $Q_{Photothermal}$ equals to Q_{loss}. Therefore, η_{PCE} can be derived by solving the differential equation (1) as follows:

$$\eta_{PCE} = \frac{hA(T-T_\infty)-Q_{sub}}{I_0(1-10^{-A(\lambda)})}, \tau = \frac{\sum mc_p}{hA} \qquad (3)$$

where τ is the time constant for the thermal system.

The Q_{sub} was negligible because the temperature change because of IR absorption of the PET substrate was less than 1.0 °C. The absorbance of the film was assumed to be four because the transmittance at 1200 nm was less than 0.5%, as shown in Figure 2(e). As a result, the calculated photothermal conversion efficiency of the hot plate-treated and oven-treated PEDOT:Tos films was 36.7% and 45.9% using equation (3). Table S1 lists the parameters used in the calculation.

Table S1. Parameters for calculating the photothermal conversion efficiency

	PET	Oven treated-PEDOT:Tos film	Hot plate treated-PEDOT:Tos film
τ (s)	-	11	12
mass(g)	0.058	0.058	0.057
T_{max} (ºC)	33.9	53.1	49.7
$T_{average}$ (ºC)	33.5	52.3	49.2
$c_p = 1200\ \mathrm{J}/kg \cdot K, T_{amb} = 24.7℃,\ I_0 = 950\ W/m^2$			
